# Renal Function and Coronary Microvascular Dysfunction in Women with Symptoms/Signs of Ischemia

**DOI:** 10.1371/journal.pone.0125374

**Published:** 2015-05-07

**Authors:** Rajesh Mohandas, Mark S. Segal, Tianyao Huo, Eileen M. Handberg, John W. Petersen, B. Delia Johnson, George Sopko, C. Noel Bairey Merz, Carl J. Pepine

**Affiliations:** 1 Nephrology and Hypertension Section, North Florida/South Georgia Veterans Health System, Gainesville, Florida, United States of America; 2 Division of Nephrology, Hypertension & Transplantation, University of Florida, Gainesville, Florida, United States of America; 3 Division of Cardiovascular Medicine, University of Florida, Gainesville, Florida, United States of America; 4 University of Pittsburgh, Pittsburgh, Pennsylvania, United States of America; 5 National Institutes of Health, Bethesda, Maryland, United States of America; 6 Barbra Streisand Women’s Heart Center, Cedars-Sinai Medical Center, Los Angeles, California, United States of America; University of Bologna, ITALY

## Abstract

**Objectives:**

Chronic kidney disease (CKD) is more prevalent among women and is associated with adverse cardiovascular events. Among women with symptoms and signs of ischemia enrolled in the Women’s Ischemia Syndrome Evaluation (WISE), a relatively high mortality rate was observed in those with no obstructive coronary artery disease. Coronary microvascular dysfunction or reduced coronary flow reserve (CFR) was a strong and independent predictor of adverse outcomes. The objective of this analysis was to determine if renal function was associated with coronary microvascular dysfunction in women with signs and symptoms of ischemia.

**Methods:**

The WISE was a multicenter, prospective, cohort study of women undergoing coronary angiography for suspected ischemia. Among 198 women with additional measurements of CFR, we determined the estimated glomerular filtration rate (eGFR) with the CKD-EPI equation. We tested the association between eGFR and CFR with regression analysis.

**Results:**

The median eGFR was 89 ml/min. The eGFR correlated with CFR (r = 0.22; *P* = 0.002). This association persisted even after covariate adjustment. Each 10-unit decrease in eGFR was associated with a 0.04-unit decrease in CFR (*P* = 0.04).There was a strong interaction between eGFR and age (*P* = 0.006): in those ≥60 years old, GFR was strongly correlated with CFR (r = 0.55; *P*<0.0001). No significant correlation was noted in those <60 years old.

**Conclusions:**

Reduced renal function was significantly associated with lower CFR in women with symptoms and signs of ischemia. Coronary microvascular dysfunction warrants additional study as a mechanism contributing to increased risk of cardiovascular events in CKD.

## Introduction

Chronic kidney disease (CKD) is associated with adverse cardiac events and excessive cardiovascular (CV) mortality[1]. Traditional risk factors for atherosclerosis do not account for the majority of risk of CV events associated with CKD [2]. Moreover, revascularization[3] or aggressive treatment of CV risk factors[4,5] have had little, or at best modest, success in preventing adverse cardiac events particularly among dialysis patients, suggesting that pathogenic mechanisms other than obstructive coronary artery disease (CAD) may contribute to excess CV mortality in those with CKD. We previously reported that in women with symptoms or signs of ischemia, even mild CKD is associated with coronary atherosclerosis[6]. In a cohort of these women, coronary microvascular dysfunction (CMD), as evidenced by a decreased coronary flow reserve (CFR) to adenosine, is a strong and independent predictor of adverse outcomes over 5.4 years of follow-up[7].

CKD is associated with inflammation, and serum levels of the acute phase reactant C reactive protein (CRP), interleukin-6 (IL-6), and serum amlyoid A (SAA) correlate with level of renal function[8] and all-cause as well as cardiovascular mortality[9]. However, the association between inflammation and microvascular disease is unclear. The pro-inflammatory cytokine IL-6 has been shown to be elevated in obese patients with decreased CFR[10]. However, in women with chest pain and no obstructive CAD, studies have not demonstrated an association between CFR and IL-6 or other inflammatory markers[11].

Interestingly, CKD is associated with microvascular dysfunction in other organ systems such as the cerebral and retinal circulation[12,13]. The role of CMD in CKD is also supported by animal models, which show that uremic mice have reduced myocardial capillary density[14]. In the setting of left ventricular hypertrophy, which commonly accompanies CKD, obstructive CAD can potentially impair vasodilatation. Pharmacological stress tests, which rely on adenosine, have decreased sensitivity in dialysis patients compared with the general population, suggesting the possibility of an impaired vasodilator response to adenosine in CKD [15].

We hypothesized that reduced renal function, determined by estimated glomerular filtration rate (eGFR), would be independently associated with a reduced CFR.

## Materials and Methods

The Women’s Ischemia Syndrome Evaluation (WISE) (clinicaltrials.gov NCT00000554), a prospective cohort study initiated by the National Heart, Lung and Blood Institute to improve the understanding of pathological mechanisms and diagnostic evaluation of ischemic heart disease in women, enrolled women with symptoms and signs of ischemia referred for coronary angiography. Details of the WISE study design have been described previously[16]. Briefly, in addition to coronary angiography, key demographic and laboratory variables were collected at baseline including assessment of CAD risk factors, blood chemistries, lipid levels, inflammatory markers, and functional capacity. Serum creatinine was measured using standard technique in the clinical laboratory at each of the four sites. Creatinine clearance (ml/min) was estimated with use of the Chronic Kidney Disease Epidemiology Collaboration (CKD-EPI) equation after decreasing creatinine levels by 5%, as recommended[17] for calibration. Lipids were measured in fasting blood plasma at the WISE lipid core laboratory (Cedars-Sinai Medical Center, Los Angeles, CA). Qualitative and quantitative CAD measures were made at the angiographic core laboratory masked to other clinical data[18]. Women with at least one ≥50% diameter stenosis were classified as having significant CAD, those with maximum stenosis ≥20% but <50% as minimal CAD, and women with <20% stenosis in all coronary arteries as no CAD. A CAD severity score was calculated by a modified Gensini index termed the WISE CAD severity score. All patients provided written informed consent, and the protocol was approved in accordance with the ethical principles outlined in the 1975 Declaration of Helsinki by the institutional review boards at each center (University of Florida, Gainesville, FL; University of Pittsburgh, Pittsburgh, PA; Cedars-Sinai Medical Center, Los Angeles, CA; and Rhode Island Hospital, Providence, RI).

### Coronary reactivity testing protocol

Coronary reactivity testing was performed in a stenosis free area of the left anterior descending coronary artery when possible, with the left circumflex artery as a secondary choice[19]. A Doppler-tipped guidewire (0.014-inch FloWire, JOMED/Cardiometrics, Mountain View, CA, now Volcano Corporation, San Diego, CA) was advanced through the diagnostic catheter, and when a stable velocity signal was obtained, baseline recordings were made. Intracoronary bolus injections of 18 mcg of adenosine (Adenocard, Fujisawa USA, Deerfield, IL), a predominantly non–endothelium-dependent microvascular dilator, was administered into the left main coronary artery. A dose of 18 mcg was chosen, since in the WISE cohort, we have previously found that CFR measurements were identical regardless of if we used low (18 mcg) or high (36 mcg) dose of adenosine[20]. At least 3 injections were done to assure a stable average peak blood flow velocity was obtained, with return to baseline velocity documented before each bolus. Pulsed-wave Doppler flow spectra were used to calculate time-averaged peak velocity (APV). Recordings were analyzed at the WISE CFR Core Lab (University of Florida) masked to all other data, and CFR was defined as the ratio of APV after adenosine to average baseline velocity just before adenosine. In prior WISE studies, this measure correlated closely (r = 0.87, *P*<0.001) with volumetric flow[18].

### Statistical analysis

There were 198 patients with measurements available for CFR and eGFR. Clinical characteristics were stratified by median renal function (eGFR ≥89 or <89 ml/min/1.73 m^2^) and CFR (≥2.5 or <2.5). Data are presented as either mean ± standard deviation for continuous variables or n(%) for categorical variables. Skewed variables are reported as medians (IQR) and transformed to log base 2 to satisfy the assumptions of homogeneity, linearity, and Gaussian distribution of errors. General linear regression was applied to compute *P*-values for continuous variables, and logistic regression for categorical variables. All *P*-values for baseline variables, except age, are age-adjusted. Normality of log_2_CFR and eGFR were tested using the Kolmogorov-Smirnov test. Pearson’s correlation was used to check univariate correlation between log_2_CFR, eGFR, and demographic factors. Multivariable linear regression analysis was conducted to identify potential predictors of CFR including history of diabetes, hypertension, dyslipidemia, metabolic syndrome, body mass index (BMI), CAD severity score, double product (heart rate X systolic blood pressure), hemoglobin, ever smoker, CRP, IL-6, SAA, age, current hormone replacement therapy, and eGFR (in unit of 10). A backward selection was employed, and covariates with *P*-value >0.2 were sequentially removed from the model. History of diabetes, hypertension, and dyslipidemia were forced into the model. All statistical analyses were conducted using SAS 9.3 (SAS Institute, Cary, NC).

## Results

### Baseline characteristics

The mean age was 55±10 years, and most women were menopausal (74%) and had multiple risk factors for CAD including hypertension (56%), hyperlipidemia (51%), metabolic syndrome (45%), diabetes (22%), and smoking (20%). The baseline characteristics categorized by renal function (eGFR ≥89 or <89 ml/min/1.73 m^2^) and CFR (≥2.5 or <2.5) are summarized in Table 1.

**Table 1:** Baseline characteristics of the study population, categorized by glomerular filtration rate (eGFR) and coronary flow reserve (CFR).

Baseline Characteristic	Total	CFR<2.5 eGFR<89 (n = 54)	CFR≥2.5 eGFR<89 (n = 48)	CFR<2.5 eGFR≥89 (n = 45)	CFR≥2.5 eGFR≥89 (n = 51)	*P*-Value*
CKD-EPI eGFR, Mean ± SD	85±19	67±17	74±10	101±8	100±9	<0.0001
**Demographics and Medical History**						
Age, years	55±10	62±10	56±9	50±9	50±8	<0.001
Post-menopausal %	74	89	77	64	65	0.86
Non-white %	18	13	15	16	27	0.14
Current HRT use %	46	36	54	47	47	0.15
SBP, mmHg	135±21	143±22	131±19	133±23	133±19	0.30
DBP, mmHg	77±11	77±10	74±12	77±8	78±12	0.79
Pulse pressure, mm Hg	59±18	66±20	56±17	56±20	55±14	0.17
Pulse, beats/min	73±12	72±11	70±13	79±11	72±11	0.81
HTN %	56	67	42	53	62	0.33
Current anti-HTN Rx %	61	70	50	62	59	0.97
Dyslipidemia %	51	62	48	53	42	0.97
Diabetes %	22	28	6	24	28	0.22
Metabolic syndrome %	45	57	39	36	46	0.17
Family history of CAD %	70	74	65	71	70	0.55
Current smoker %	20	13	19	24	26	0.94
Ever smoker %	57	63	50	53	62	0.66
DASI, medians (Q1,Q3)	14(7,25)	10(5,16)	24(11,32)	13(7,24)	12(7,25)	0.38
**Laboratory Data**						
Total cholesterol, mg/dl	188±45	194±48	183±42	184±39	189±49	0.94
Triglycerides, mg/dl	144±140	153±115	133±105	130±94	160±209	0.98
HDL, mg/dl	51±13	52±11	50±12	52±12	51±15	0.77
LDL, mg/dl (calculated)	111±39	119±45	106±34	109±35	111±42	0.57
Hemoglobin, g/dl	13.0±1.4	13.0±1.7	13.4±1.1	12.9±1.1	12.6±1.4	0.15
Creatinine, mg/dl	0.79±0.22	0.96±0.30	0.87±0.11	0.64±0.10	0.66±0.10	<0.0001
**Inflammatory Markers**						
Hs-CRP, mg/dl, medians (Q1, Q3)	(0.15,0.88)	(0.20,0.80)	(011,0.63)	(0.20,0.98)	(0.16,0.95)	0.19
IL6, pg/ml, medians (Q1, Q3)	(1.71,5.26)	(1.69,5.75)	(1.87,4.84)	(1.82,4.65)	(1.65,4.51)	0.94
SAA, mg/dl, medians (Q1, Q3)	(0.30,0.94)	(0.33, 1.20)	(0.24,0.58)	(0.33,0.95)	(0.35,0.90)	0.73
**Angiographic Data**						
CAD(≥ 50% stenosis)%	18	36	17	11	14	0.08
CAD severity score, medians (Q1, Q3)	6(5,9)	9(5,14)	5(5,8)	5(5,8)	5(5,9)	0.16
CFR, medians (Q1, Q3)	2.5(2.1,3)	2.0(1.7,2.3)	3.0(2.6,3.4)	2.2(1.9,2.3)	2.9(2.6,3.3)	<0.0001

CKD-EPI = Chronic Kidney Disease Epidemiology Collaboration equation, eGFR = estimated glomerular filtration rate, HRT = hormone replacement therapy, SBP = systolic blood pressure, DBP = diastolic blood pressure, HTN = hypertension, CAD = coronary artery disease, DASI = Duke Activity Status Index, HDL = high density lipoprotein, LDL = low density lipoprotein, Hs-CRP = high-sensitivity C-reactive protein, Q1 = 25^th^ percentile, Q3 = 75^th^ percentile, IL-6 = interleukin 6, SAA = serum amyloid A. *P*-values represent the comparison between four GFR/CFR combination groups. *P*-values for all variables except age were adjusted for age.

### Renal function and CFR

**Fig 1:**
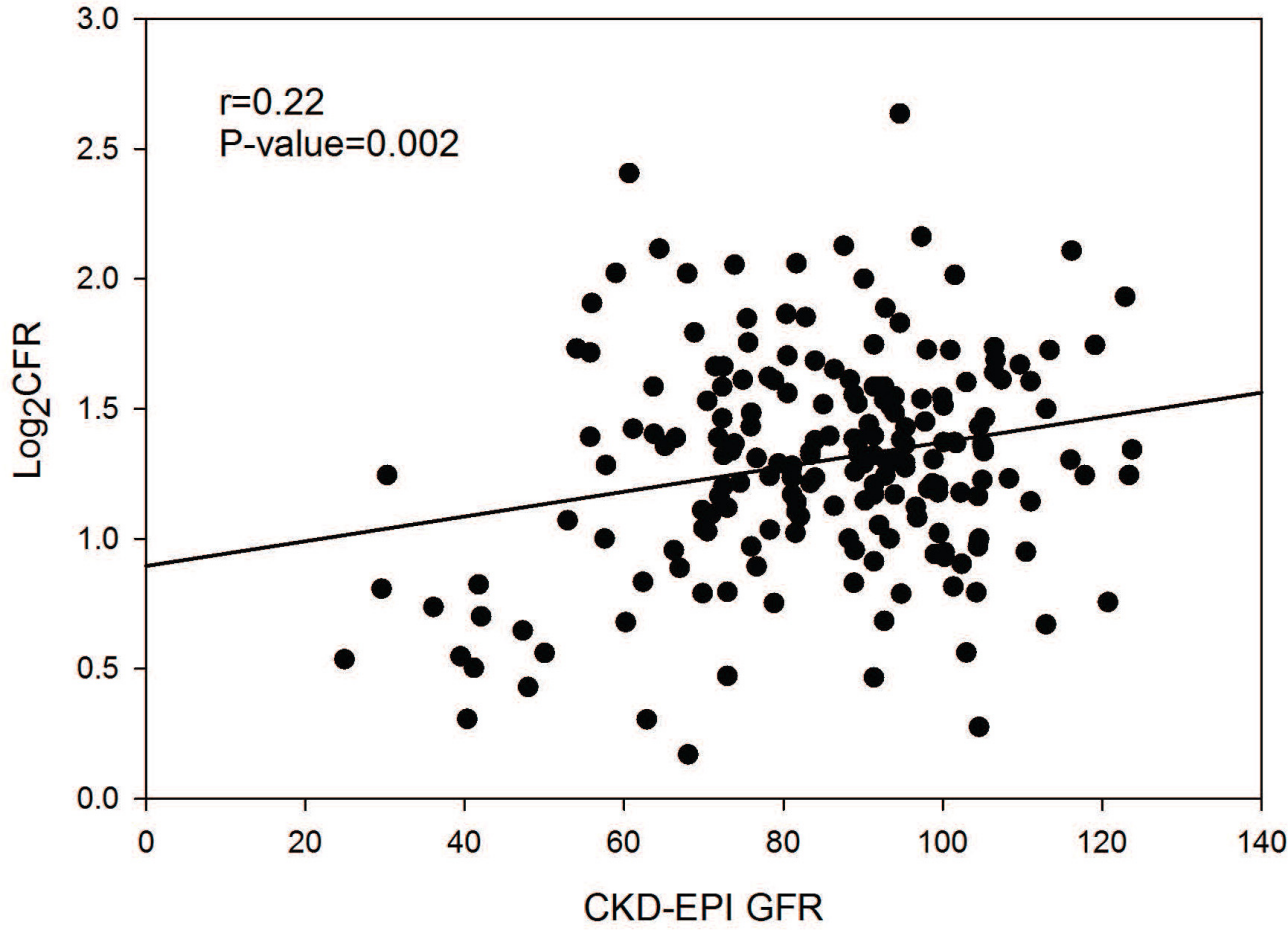
Linear Regression of CFR and eGFR. Coronary flow reserve (CFR) was log transformed. Estimated glomerular filtration rate (eGFR) was determined using the Chronic Kidney Disease Epidemiology Collaboration (CKD-EPI) equation.

In univariate analysis, renal function, eGFR, was significantly correlated with CFR (r = 0.22, *P* = 0.002), as shown in Fig 1. The association persisted even when adjusted for age, diabetes, hypertension, dyslipidemia, double product, BMI, severity of obstructive CAD, and current hormone replacement therapy (*P* = 0.0003, model R^2^ = 0.18). No other variables were significant independent predictors of CFR. Results of the multivariable regression are summarized in Table 2.

**Table 2:** Independent predictors of coronary flow reserve (CFR).

	Model 1 (R^2^ = 0.18)		Model 2 (R^2^ = 0.22)	
	β (SE)	P-value (add in order)	β (SE)	P-value (add in order)
Age	-0.05(0.04)	0.0003	0.03(0.05)	0.0003
Diabetes	0.02(0.09)	0.52	-0.004(0.09)	0.52
Hypertension	-0.08(0.07)	0.31	-0.08(0.07)	0.29
Dyslipidemia	-0.04(0.07)	0.52	-0.04(0.07)	0.51
HR×SBP	-0.04(0.02)	0.041	-0.03(0.02)	0.037
BMI	0.008(0.005)	0.15	0.006(0.005)	0.14
CAD score	-0.009(0.005)	0.06	-0.01(0.005)	0.05
Current HRT	0.13(0.06)	0.047	0.12(0.06)	0.042
eGFR	0.04(0.02)	0.040	0.01(0.02)	0.036
Interaction of eGFR and Age	-	-	0.05(0.02)	0.006

SE = standard error, HR = heart rate, SBP = systolic blood pressure, BMI = body mass index, CAD = coronary artery disease, HRT = hormone replacement therapy, eGFR = glomerular filtration rate ml/min/per 1.73 m^2^. Age and eGFR were divided by 10 and double product by 1000 for consistency.

### Inflammatory markers, eGFR, and CFR

When stratified by eGFR and CFR, in women with renal dysfunction, CRP, IL-6, and SAA were higher in those with low CFR compared with those with normal CFR. However, analysis of variance adjusted for age showed no significant differences between groups (Table 1). In the multivariable regression model, inflammatory markers CRP, IL-6, and SAA did not contribute significantly to the model.

### Age, renal function, and CFR

**Fig 2:**
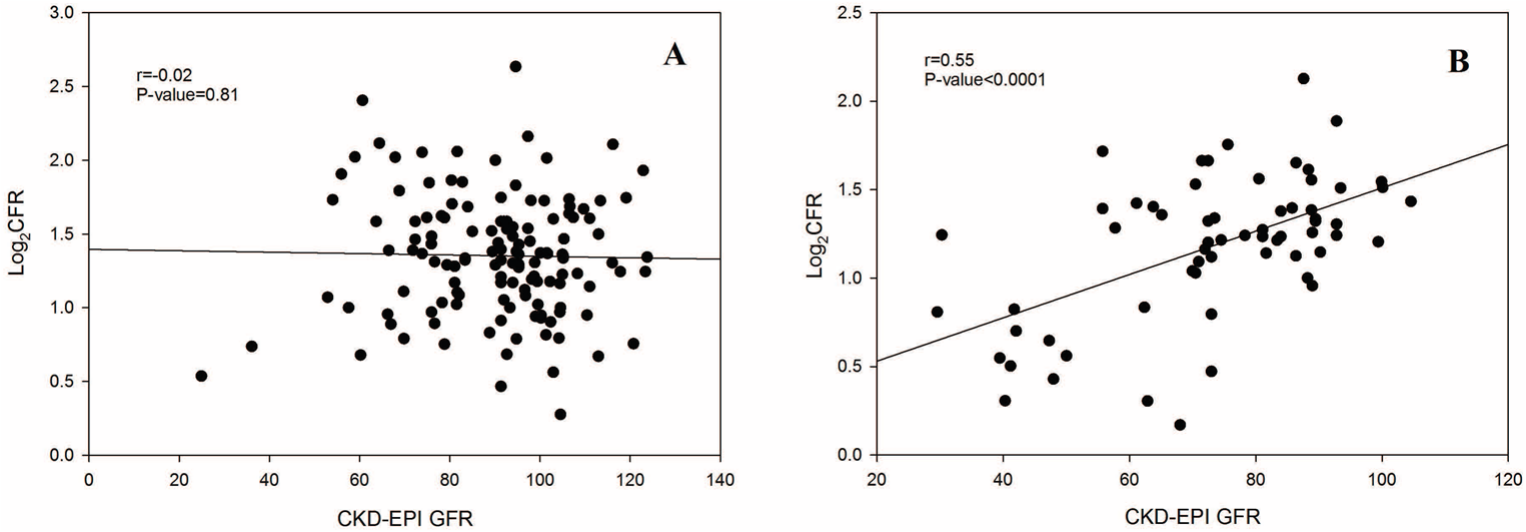
Scatter plot of eGFR and Log_2_CFR in women (A) <60 years of age (n = 135) and (B) ≥60 years of age (n = 63). C oronary flow reserve (CFR) was log transformed. Estimated glomerular filtration rate (eGFR) was determined using the Chronic Kidney Disease Epidemiology Collaboration (CKD-EPI) equation.

When age was included in the model, eGFR remained an independent predictor of CFR (r = 0.22, *P* = 0.0001) even though age and renal function showed a significant interaction (*P* = 0.006). When age was dichotomized as < or ≥60 years, 42% of women with eGFR<89 ml/min/1.73 m^2^ and 48% of those with eGFR ≥89 ml/min/1.73 m^2^ who were <60 years old had a low CFR (<2.5). However, in those ≥60 years old, 63% of those with eGFR <89 ml/min/1.73 m^2^ had a low CFR compared with only 36% of those with eGFR ≥89 ml/min/1.73 m^2^. eGFR and CFR showed a linear correlation among those ≥60 years old (r = 0.55, *P*<0.0001), while there was no significant correlation in those <60 years old (r = 0.02, *P* = 0.81) (Fig 2). The interaction of age and renal function persisted even when hormone replacement therapy was included as a covariate.

## Discussion

Studies examining the association of CFR with renal function have shown conflicting results. A study of 22 non-diabetic subjects found no significant differences in CFR between individuals with moderate to severe CKD and 10 healthy controls[21]. However, this was a relatively small sample of mostly men (66%), and even the apparently healthy controls had an average eGFR of 76±5 ml/min/1.73 m^2^, which is below normal. Others have found an association between CFR and renal function. Charytan et al. assessed CFR by positron emission tomography in 435 non-diabetic individuals. Although baseline CFR was significantly associated with eGFR, no significant association was found after adjusting for age and hypertension status, but on longitudinal follow up a decline in eGFR was a strong and independent predictor of a decrease in CFR[22]. However, Charytan et al. estimated CFR using intravenous adenosine with positron imaging, and coronary angiographic data were not available. Although overall the patients were mostly male (74%), there were significant sex differences in the subgroups, with 51% women in the severe CKD group but only 27% in mild CKD and 16% in healthy controls. Thus it is not clear how their results apply to women, particularly those without significant obstructive CAD.

Chade et al. assessed CFR using intracoronary adenosine in 605 patients without significant obstructive CAD[23]. Patients with eGFR ≥60 ml/min/1.73 m^2^ had higher CFR compared to those with eGFR <60 ml/min/1.73 m^2^. CFR was lower in women, the elderly, and those with hypertension. Multiple logistic regression analysis adjusted for age, sex, and hypertension showed that the association of renal function and CFR persisted. But when subjects were dichotomized into groups with eGFR ≥60 and <60 ml/min/1.73 m^2^, there was no discernible effect of milder degrees of renal failure on CFR, particularly in women. Bezante et al. suggested that an eGFR of <60 ml/min/1.73 m^2^ conferred a seven-fold higher risk of having an impaired CFR[24], but there were only 7 women with CKD.

Our study, the only one focusing solely on women, contained the largest number of women with coronary angiographic data, and had a prospectively defined protocol requiring that angiograms and CFR recordings be read by core labs masked to all other patient data. Determination of CMD was by direct coronary blood flow measurements in response to intracoronary adenosine to measure CFR. We found that even among women with only mild renal dysfunction, CFR is linearly associated with eGFR, with each 10-unit decrease in eGFR being associated with a 0.02-unit decrease in CFR (*P* = 0.004). We have previously shown in the WISE cohort that clinical and demographic variables explain only 16% of the variance in CFR and are not independently associated with CFR. eGFR on the other hand is significantly associated with CFR, even after controlling for multiple covariates including diabetes, hypertension, dyslipidemia and severity of obstructive coronary artery disease.

An important confounder is that CKD is more prevalent with aging[25]. Likewise CMD is more frequent in older individuals, particularly those ≥60 years old[25,26]. As expected, when age is included as a covariate in models where GFR is estimated using equations as opposed to directly measured, the association between GFR and cardiovascular events is greatly reduced[27]. Importantly, in our model eGFR was associated with CFR even when controlled for age, validating the strength of the association. There was a significant interaction between age and renal function—in those ≥60 years old there was a strong linear correlation between eGFR and CFR which was not present in those <60 years old. Further mechanistic studies are critical to determine whether coronary microvascular and renal dysfunction are due to aging, per se, or due to a common, as yet unidentified, pathological process.

A low CFR has been associated with inflammation. In discordant male twins, Vaccarino et al. found that reduced CFR on PET scan was associated with elevated levels of inflammatory markers[28]. A decreased CFR has also been demonstrated in inflammatory disorders such as rheumatoid arthritis[29] and lupus[30]. Interestingly, in our exclusively female cohort inflammatory markers such as CRP, IL-6, and SAA were not associated with renal function and did not add to the ability of eGFR to predict CFR. This suggests that in women with CMD, CKD and decreased CFR are not a consequence of inflammation. CKD is often associated with hypertension and left ventricular hypertrophy (LVH). Decreased CFR has been demonstrated in conditions accompanied by LVH such as aortic stenosis[31] and Fabry’s disease[32]. In the WISE cohort, characterized by mild renal dysfunction and very little ventricular hypertrophy, we have previously shown that LVH does not predict CFR.

### Study limitations

Although our study showed a statistically significant relation between CFR and GFR, renal function only explains 4% of the variance in CFR overall (r = 0.21) and 30% in those over the age of 60 years (r = 0.55). This is at least in part because the WISE cohort included women who were referred for coronary angiography and thus excluded those with severe renal dysfunction. This limits generalization of results to other populations. We did not assess proteinuria at baseline or at follow up. Recent data suggest that presence of albuminuria would better stratify those at risk for vascular events, especially among those with milder degrees of CKD[33]. We did not have longitudinal assessment of renal function. This is particularly relevant since some studies have suggested that CFR correlates with changes in renal function[22]. Although our limited numbers precluded subgroup and outcome analysis, we have previously shown that CFR was the most important predictor of major adverse outcomes in a larger WISE cohort with 5.4 years follow-up[7]. This also held true among the women without obstructive CAD. The CFR significantly improved prediction of adverse outcomes over angiographic CAD severity and other risk conditions. Recent studies have suggested that CFR might predict those at high risk of CAD events in patients with CKD[34,35].

## Conclusion

Our results demonstrate that renal function estimated by eGFR is significantly associated with CFR, and even mild decline in renal function is associated with CMD. Treatment modalities directed at traditional risk factors for atherosclerosis have had little impact in reducing mortality and morbidity in patients with CKD. CMD deserves additional study as a contributor to increased cardiovascular morbidity and mortality associated with CKD. Understanding the mechanistic basis of CMD in women with CKD has the potential to identify targets for novel therapeutics and to improve prognosis in a high-risk cohort for whom conventional therapies have been uniformly disappointing.
